# Editorial: Advancements in neural learning control for enhanced multi-robot coordination

**DOI:** 10.3389/frobt.2025.1731356

**Published:** 2025-11-21

**Authors:** Shude He, Shi-Lu Dai, Chengzhi Yuan, Haotian Shi

**Affiliations:** 1 Mechanical and Electrical Engineering, Guangzhou University, Guangzhou, China; 2 Key Laboratory of Marine Environmental Survey Technology and Application, Ministry of Natural Resources, Guangzhou, China; 3 School of Automation Science and Engineering, South China University of Technology, Guangzhou, China; 4 Department of Mechanical, Industrial and Systems Engineering, University of Rhode Island, Kingston, RI, United States; 5 School of Electronics and Communication Engineering, Guangzhou University, Guangzhou, China

**Keywords:** neural network control, learning control, dynamic learning, reinforcement learning, adaptive control, robotics, autonomous vehicles, multi robotics

## 1 Summary

Multi-robot systems are increasingly deployed in complex, dynamic environments such as environmental monitoring, industrial automation, and search-and-rescue missions. The coordination of such systems poses significant challenges, including path planning, real-time adaptation, and efficient resource utilization. Recent advances in neural learning control encompassing reinforcement learning, deterministic learning, deep learning, and adaptive algorithms have opened new frontiers for enhancing the autonomy, robustness, and scalability of multi-robot coordination. This Research Topic brings together cutting-edge research that leverages neural learning techniques to address these challenges, with a focus on real-world applicability and system-level intelligence.

## 2 Contents of the Research Topic

This Research Topic aims to provide a platform for researchers to further investigate related issues of neural learning control for enhanced multi-robot coordination and publish their latest research achievements. Organized under the section “Robot Learning and Evolution”, this Research Topic has published four articles. All the accepted papers are summarized as follows.

The paper “Path planning of industrial robots based on the adaptive field cooperative sampling algorithm” by Zhuang et al. introduces the adaptive field cooperative sampling (AFCS) algorithm, which integrates Rapidly-exploring Random Trees (RRT) with an improved artificial potential field method. By incorporating environmental complexity functions and cooperative expansion strategies, the algorithm significantly enhances path quality, planning efficiency, and adaptability in complex industrial settings. Simulations and real-world experiments on a ROKAE robot validate its superiority over traditional RRT variants.

The paper “ModuCLIP: Multi-scale CLIP framework for predicting foundation pit deformation in multi-modal robotic systems” by Lin et al. proposes a novel fusion of contrastive learning and multi-scale feature extraction for real-time deformation prediction. The framework integrates visual, textual, and sensor data, demonstrating state-of-the-art performance in accuracy and generalization. This work highlights the potential of cross-modal neural models in safety-critical robotic monitoring systems.

The paper “Reinforcement learning-based dynamic field exploration and reconstruction using multi-robot systems” by Lu et al. combines proximal policy optimization with K-means clustering for efficient exploration and reconstruction of dynamic diffusion fields. The two-mode strategy alternating between source mapping and field exploration ensures rapid detection and accurate parameter estimation, validated through both simulations and lab experiments.

The paper “Adaptive formation learning control for cooperative AUVs under complete uncertainty” by Jandaghi et al. proposes a novel two-layer control framework. By integrating a cooperative estimator for distributed state estimation with a decentralized deterministic learning controller using radial basis function neural networks, the method achieves precise formation tracking without prior knowledge of robot parameters or environmental conditions. A key innovation is the system’s ability to learn and store dynamic knowledge as constant neural weights, enabling efficient reuse after system restarts without retraining. Comprehensive simulations demonstrate the framework’s stability, resilience, and superior adaptability in complex underwater scenarios.

The four studies in this Research Topic collectively advance the paradigm of neural learning control for multi-robot coordination by addressing domain-specific challenges while uncovering universal insights: 1. Learning from interaction: Reinforcement learning (Lu et al.) and Deterministic learning (Jandaghi et al.) enable robots to adapt to dynamic environments without pre-defined models—key for tasks like environmental monitoring and underwater exploration. 2. Multi-modal fusion: Contrastive learning (Lin et al.) breaks down data silos in multi-robot systems, integrating visual, textual, and sensor data to improve prediction accuracy in geotechnical and environmental tasks. 3. Efficiency-generalization balance: AFCS (Zhuang et al.) demonstrates that adaptive optimization can reduce redundancy in path planning, laying the groundwork for scalable multi-robot industrial coordination.

## 3 Conclusion

In this special Research Topic, we explore the cutting-edge domain of neural learning control and its transformative impact on multi-robot coordination. Each article represents a significant contribution to advancing the intelligence, adaptability, and scalability of robotic systems operating in complex environments. We extend our sincere appreciation to all authors for their valuable research, which pushes the boundaries of what’s possible in multi-robot coordination. As we stand at the forefront of a new era in intelligent robotics, these innovations pave the way for more sophisticated applications in cooperative intelligence, industrial automation, and beyond. We invite readers to explore the significant research achievements within these pages and hope this Research Topic inspires new breakthroughs in neural learning-driven multi-robot systems that will ultimately lead to safer, more efficient, and more autonomous robotic solutions for our future.

